# The ‘Merry-Go-Round’ of Habitual Relapse: A Qualitative Study of Relapse in Electronic Gaming Machine Problem Gambling

**DOI:** 10.3390/ijerph16162858

**Published:** 2019-08-10

**Authors:** Jane Oakes, Rene Pols, Sharon Lawn

**Affiliations:** 1 PsychMed, Wellbeing and Recovery Research Institute—WARRI, Monash Addiction Research Centre, Eastern Health Clinical School, Monash University, 5000 Adelaide, Australia; 2 Flinders Human Behaviour and Health Research Unit, Department of Psychiatry, Flinders University, 5042 Adelaide, Australia

**Keywords:** problem gambling, relapse, focus group, in-depth interviews, despair, gambling harms, negative emotions

## Abstract

Background: Our understanding of gambling relapse is limited despite the damaging consequences affecting many aspects of the gambler’s life. Paradoxically, regardless of these negative consequences problem gamblers (PGs) continue to relapse, seemingly unable to stop this cycle of harm. This paper addresses the phenomenon of repeated gambling relapse shedding some insights into why gamblers continue to relapse. Methods: The study comprised of (*n* = 54) participants purposefully selected who participated in either 1 of 5 focus groups (*n* = 35) or in-depth interviews (*n* = 19). The new knowledge obtained was from PGs, significant others, and workers with direct experience of gambling relapse. Interview recordings were analysed using thematic, textual analysis. Results: The avoidance of negative emotions from the consequences of the destructive behaviour associated with repeated relapse leads to a hopeless “merry-go-round”. Once on this “merry go round”, relapse becomes a habitual way of life where behaviour change and learning from the devastation of a gambling relapse is challenging. Exiting this cycle means PGs must face the consequences of their gambling which for many is overwhelming, and relapse is a way to avoid despair. Conclusions: These findings provide insights into relapse which has implications for gamblers seeking treatment, assessment and treatment “drop-outs”.

## 1. Introduction

Problem gamblers (PG) experience high levels of depression, anxiety, suicidal ideation, dissociation [1], and significant harms [2,3]. In one study, the total burden of harms to gamblers was found to be higher than common health conditions such as diabetes and arthritis and, possibly, the levels of anxiety and depressive disorders also. In addition, financial pressures damaged relationships and caused emotional and psychological distress [2]. Furthermore, a significant proportion of PGs relapse and, in the majority of these gamblers, relapse will likely result in uncontrolled gambling behaviour with many damaging consequences for the person and those around them [4,5]. Despite these harms, little is understood about relapse in problem gambling [6] and the current literature has limited explanatory power for this complex phenomenon to date. Furthermore, the majority of studies exploring relapse in problem gambling have focused mainly on single factors in isolation [7]. These factors do not account for the complex interactions of facilitatory and protective factors that affect relapse in problem gambling. For example, when a gambler enters a high-risk situation or experiences a physiological response such as an urge to gamble, the risk of gambling depends on the individual’s coping skills, their personality and biological variables, or erroneous cognitions and expectations about gambling. The authors highlighted that future research needs to examine the interrelationship between these psychological, social and biological factor [8].

Many studies of PGs have explored factors associated with treatment drop-out and relapse and provide an understanding about precipitating factors. For example, factors which can increase the odds of a gambling relapse include a lifelong history of a mood disorder, an alcohol abuse diagnosis, and when support ended during treatment follow-up [9]. High levels of distress, poor quality of life [10,11], cognitive factors such as cognitions about winning, and emotional factors. Men attributed gambling relapses to the need to make money, unstructured time, and boredom. Women reported gambling more often to deal with negative emotions or stressful life situations. The ‘need to fit in’ was associated with minor relapses [5].

Treatment studies can also provide an understanding of gambling relapse. For example, gamblers with an earlier age of onset of gambling problems, a higher level of novelty seeking, and low self-directedness [12], reduced motivation to change, distress, higher levels of obsessive-compulsive symptomatology, or an inadequate response to treatment [13] have been associated with a higher risk of treatment drop-out and relapse. In an earlier study most treatment failures were associated with reduced satisfaction with treatment, heavy drinking and a high level of neuroticism as a personality variable. These studies have identified some critical risk factors for relapse but have failed to understand how these factors can interact and impact on relapse [14]. Importantly, Echeburua et al. (2000) highlighted the importance of teaching people to identify high-risk situations and strategies to cope with those situations [15].

Gambling behaviour is a complex process involving factors that together can increase a gambler’s vulnerability to relapse. This process fits with the conceptualisation of relapse in alcohol dependence [7,8]. These authors suggest that relapse, as in the case of alcohol, is not a linear process, but rather relapse is a complex process where changes in one risk factor may cause increased cravings, positive outcome expectancies, and negative affect, often leading to a significant relapse. This devastating experience can lead to the realisation that regaining abstinence will not be straightforward [8] Furthermore, the disease model of addiction suggests that due to physiological and genetic predisposing factors, a person cannot exercise control over their drug use. This belief implies that the person is not able to control their drug use and releases the person from accepting personal responsibility for their addiction (Marlatt and Gordon, 1985). However, Marlatt and Gordon’s (1985) relapse prevention (RP) model is based on social learning principles, which highlights the person’s need for participation and responsibility without taking on the blame. The RP model proposed by Marlatt and Gordon (1985) suggests the importance of developing individual relapse road maps that take into consideration the immediate relapse determinants. For example, their high-risk situations, coping skills, outcome expectancies, the abstinence violation effect, and covert antecedents which include lifestyle factors, urges and cravings. This RP model encompasses targeted interventions to enhance a person’s coping skills in high-risk situations. For, example increasing the person’s self-efficacy, eliminating their myths regarding the effects of alcohol, helping them to manage lapses, and restructuring their perceptions of the relapse process. Other strategies include balancing the client’s lifestyle and helping them develop positive addictions, employing stimulus control techniques, and ways to manage their urges [16]. There are multiple causes of relapse which highlights the need for such a comprehensive program that specifically addresses relapse prevention, for example, combining classical cue exposure techniques such as exposure and response prevention with coping-skill training in the clients’ real or simulated high-risk situations such as drug cues [17]. The primary purpose of the present study was to gain an understanding of the phenomenon of repeated gambling relapse, for example, examining why PGs continue to relapse despite the ongoing harms that continue to accumulate as a result of this behaviour. A qualitative approach, combining focus group (FG) methodology and face-to-face in-depth interviews, provided a rich understanding of cognitive processes involved in problem gambling and repeated relapse in this under-researched area.

## 2. Materials and Methods

The use of a qualitative methodology enabled the researchers to listen to and interpret what participants had said about their experiences of electronic gaming machine (EGM) problem gambling [18]. EGMs are comparable to slot machines [19], poker machines’ [20] ‘video poker’ [21] or ‘fixed-odds betting terminals’ [22]. The idea of combining more than one type of qualitative approach can bring a particular type of insight into an enquiry based on the personal experiences and views of individuals. For example, focus groups used in the initial stages of an investigation raised and explored relevant issues that were further explored through in-depth interviews, discussing findings at a more strategic level [23]. Focus groups are a method of choice when studying participants’ lives, underlying beliefs, opinions held, or for constituting a social context acquired by direct observation [24] Therefore, the first phase of this study used focus groups to obtain an initial description of the relapse processes. The second phase employed in-depth interviews to provide a further, deeper understanding of relapse, as the researcher and participants engaged in a dialogue in which the researcher could probe interesting and important areas that had been discovered in the focus group component of the study. Data from the in-depth interviews strengthened the focus group findings with rich data about the direct experiences of participants with regards to EGM problem gambling [25].

Participant recruitment began following ethics approval. The decision to recruit participants was based on a qualitative methodology, which uses small sample sizes selected purposefully to permit enquiry into, and to understand, a phenomenon in great depth. Purposive sample selection [23] was, therefore, used for this study in order to examine relapse from multiple perspectives, and to ascertain how the process of relapse occurs. In addition, a small number of participants who are “information rich” can offer useful manifestations of the phenomenon of interest, providing the power behind the in-depth understanding of the phenomenon being studied [26]. The gambling participants who were recruited for this study had self-identified as gamblers, rather than being identified by fulfilling criteria through diagnostic screening tools. This enabled a broad range of PGs to share their stories. The study comprised (*n* = 54) participants purposefully selected who participated in either 1 of 5 focus groups (*n* = 35) or an in-depth interview (*n* = 19). The new knowledge obtained in this study was acquired from PGs, significant others (for example, spouse or other family members), and workers with direct experience of gambling relapse. Focus group participants were purposely selected from gamblers attending support services for those who have struggled with EGM problem gambling in South Australia. Participants were recruited from non-government agencies such as Pokies Anonymous, gambling help services, hotels, and a number of mental health services in South Australia, for the in-depth interviews. Pokies Anonymous is similar to Gamblers Anonymous [27]. The researchers reviewed the relevant literature to help direct the questions and topics to be examined in the focus group discussions [28]. A structured interview guide was important to direct the focus group discussions towards the main themes the researcher expected to focus on [28]. These questions covered: psychological factors (cognitive, affective, personality); psychobiological factors; social and environmental factors; and, treatment. Interview questions were developed in consultation with the literature, taking into consideration what was established in the focus group study [29].

All interviews were audio-recorded, transcribed and analysed using thematic analysis, including open coding, axial coding, constant comparative analysis, a systematic generation of theories from the data that contains both inductive and deductive thinking, and synthesis of the data [30].

The process of comparative reflection of the transcribed focus group and interview data against each other, the literature, and from the ongoing discussions between the researchers, continued until no further new data or themes emerged [31]. This ensured that saturation of the data had been achieved. An external auditor (SL) reviewed the fidelity of the methodology and analytic process, confirming a clear audit trail [32]. The Flinders University Health Research Ethics Committee (No. 3948) approved this study.

Data is described using pseudonyms (for participants) to display de-identified participants’ quotations, identified by whether participants were recruited for focus groups or in-depth interviews (I). The following definition for relapse was used for this study as it was observable and measurable and could generalise to the differing problem gambling presentations. “Relapse is the re-emergence of gambling that may cause harm to the individual, significant others, or the community after a period of abstinence or controlled gambling” [6] (p. 16).

## 3. Results

**Figure 1:**
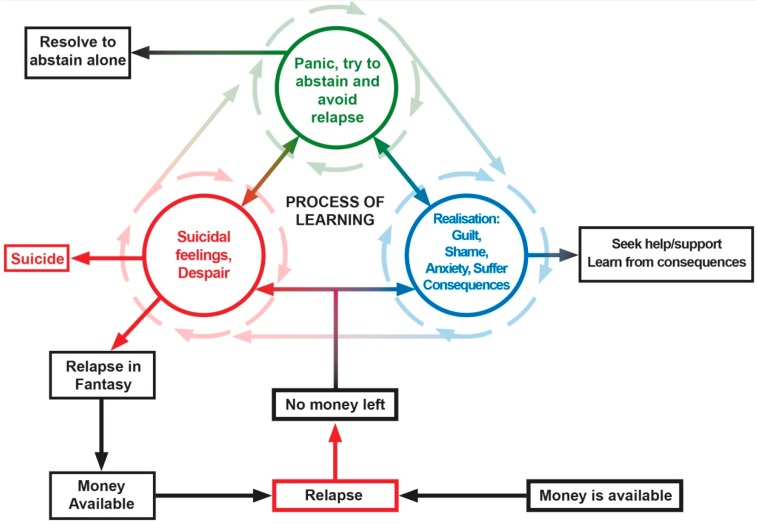
The ‘Merry-Go-Round’ of Relapse.

It was evident that PGs were trapped on what appeared to be a “merry go round” of relapse, where learning from the consequential harm seemed to be impaired because it was so difficult for them to leave. This complex of competing emotions and cognitions are pictorially and conceptually summarised in Figure 1. These experiences occurred cumulatively and longitudinally over time, but most of the PG subjects had experienced these states of mind and gambling cognitions at one time or another.

### 3.1. ‘Merry-Go-Round’ of Repeated Harms

John described feeling extremely emotional after losing all his money gambling, but this distress was pushed aside, and excuses were made to gamble once again. It appears that critical thought was not possible; all harms were forgotten; instead, John decided to focus, almost without thinking, fantasysing about the next possible gambling episode and about winning:

“I mean the full-blown emotion of losing all of that would subside after a couple of days then I felt okay again. So, guilt and all that would subside and then I would start again, and I always had a bit of an excuse to go in” (Treatment Seeker: 1 aged 48).

The deliberate and habitual nature of relapse was described by Nanette who repeatedly chose to gamble while numbing herself from the distressing emotions of past losses as she went on “automatic”, believing each time that it would be different, and she would win:

“Just the same as always. You just automatically went back because once you got in there, the same situation occurred; you just felt that this time it’s going to be different, I’m going to win, but it hardly ever happened” (Pokies Anonymous Member: I, aged 72).

Nick was trapped on the “merry-go-round” where he gambled in quick succession. For example, after a significant loss, he returned home aware of the financial harm he had caused himself. This realisation was exceptionally distressing for Nick, but he pushed these emotions aside by returning to gamble and the harms were forgotten again; whenever immersed in gambling:

“I blew a heap of damn money, and I said, ‘this is no good’. Had a bit of tea and then I went back. While you’re doing it—it’s feeling good when you’re doing it, but it’s when you get home at night time when you come home after you’ve blown all your bloody money it’s a terrible feeling” (Pokies Anonymous Member: I, aged 65).

Annie continued on the gambling “merry-go-round”, enduring the same harms each week. Her ability to make rational decisions and exercise the will to stop gambling were not possible, being in a constant state of hopefulness about winning:

“Every week it was the same thing, the whole pension, back to the churches, back to the – it was horrible” (Pokies Anonymous Member: I, aged 53).

### 3.2. Loss of Control

Loss of control was described by all PGs on the “merry go round”. For example, Lisa’s judgement was impaired because she could not remember the harm or the predetermined financial limit she had set. She admitted that it was not possible for her to think logically when gambling, and her only option at this time was not to gamble at all:

“Well, all sensibility goes out the window, literally, and you don’t think about it then. If I go in and put one dollar in the poker machine—I went through a stage where I thought ‘well, I’ll only put $20 in, no more’; that doesn’t happen. You can’t do it. You either have to not play them at all, or $20 becomes $50, and $50 becomes $100” (Pokies Anonymous Member: I, aged 60).

A non-government worker (NGO) highlighted that she saw clients, for example, who loved the buzz associated with the urge to gamble, but this soon went, and they no longer liked the feeling, but became helpless to resist and relapsed:

“You will get a client who says, I still love it, it gives me a buzz… but generally people don’t, they say I hate them. So, by the time they come to someone like yourself, they are still engaging in that type of gambling behaviour, but they dislike what they do, there’s the addiction side of it they just can’t break” (NGO worker: FG: aged 40–55).

### 3.3. Deferral of Relapse

As the gambler focused attention on winning during the next gambling episode, very soon and in some cases immediately after losing, they were able to avoid experiencing negative emotions such as shame, guilt, and self-loathing associated with the consequences of their gambling. Therefore, this repeated relapse cycle gave the gambler minimal time to experience any impacts related to their gambling. Barb admitted that her friends had seen her try to abstain and then relapse in the past, and they would point out to her that she was beginning the relapse cycle again:

“Yeah, and they would say to me ‘Oh you’re not on that’—because I’ve done this a few times, I might add, and my very dear friends would say to me ‘Oh no, you’re not going through that stage again?’ and I’d say ‘Yeah,’ but it didn’t bother me” (Non-Treatment Seeker: I, aged 78).

Annie remembers how she could not wait to gamble and chase her losses. Despite having lost all accessible money to gamble, she would start to plan her next relapse seeking the win that will make it all right again, indicating an inability to think critically:

“You can’t wait for your next pay and try and get that back” (Pokies Anonymous Member: I, aged 53).

### 3.4. Negative Emotions

The excitement and distraction obtained from the fantasy of winning provided an escape from experiencing negative emotions. For example, Nanette was suffering significant grief from the loss of her husband. She admitted that the only place she could go and escape her overwhelming grief was to the hotel to play the Pokies. For Nanette, entering the gambling “merry-go-round” enabled her to have some relief from her suffering, making it difficult for her to stop the cycle:

“All you feel like doing is crying all day. You know you can’t keep doing that, you’ve got to move. The only place you can go is to the Pokies” (Pokies Anonymous Member: I, aged 65).

Kath described how her worries gradually went away as she became “fixed” on the EGM screen as if it were a drug and she had no control. Her total focus on gambling had allowed her to forget her anxiety and depression in the short-term:

“Yeah, I think sometimes you go, and you’re sort of fixated on the screen, and your worries seem to sort of just dissipate…and it’s, I guess, a cop-out for your anxiety and your worry. How could you put it? It’s like—I don’t know whether it’s a drug, that when you sit in front of a machine, and you might be depressed or anxious, there’s nothing else worrying you and you’re just totally fixated on the screen” (Non-Treatment Seeker I, aged 48).

### 3.5. Despair from Being Trapped on the ‘Merry-Go-Round’

Many participants described the overwhelming despair they experienced as they continued to gamble despite the harms. Annie revealed how distressed she felt each time she gambled; but, despite the despair, shame and guilt she could not stop herself from being in the vicious cycle of gambling:

“I was afraid, I was ashamed, I was really embarrassed. I left with a big fat blanket of guilt, horrific, horrific feeling. What have I done again?” (Pokies Anonymous Member: I, aged 53).

Nanette felt sick each time she promised herself not to gamble again:

“You felt sick after promising yourself never, ever again and you still go back and do it again” (Pokies Anonymous Member: I, aged 65).

### 3.6. The ‘Mythical Win’

Participants talked about their belief in a “mythical win” where the EGM would provide them with a win that would give a solution to their problems.

For example, Barb had a belief that she would win the jackpot, and planned how she would spend it:

“You’re going to win the money back and saying one day you’re going to hit that jackpot and it’s going to be so big you’ll be able to do this and do that, you know” (Non-treatment seeker: I, aged 78)

Nick was sure he could win some money just like other gamblers and with this myth he fantasised that he would pay off some bills and buy himself something extra:

“You just want to get out, get some more money out and try and win back what you’ve lost. think sometimes you’re sort of hoping that you can actually win some money, whether to pay extra bills off or to buy extra stuff. You think you’ve seen other people win money, why can’t I?” (Pokies Anonymous Member: I, aged 65).

As Kath gambled with her husband, she was excited as she imagined with her winnings she would be replacing her old carpet and buying lots of different things she needed which kept her gambling:

“Well you’re excited about it because you’re sitting there chit chatting about it and you think somebody’s going to win, it could be us, and then we’ll get a new carpet and then we’ll get this and then we’ll get something else so you think oh well another $50 will just do it and off you go and get another $50” (Non-Treatment Seeker I, aged 48).

The “mythical win” is different from the normal win for some it is not excitement about winning and what you would do with the money. For these gamblers it is a win that will expunge the guilt and harm done by their gambling behaviour. It is about reparation and allows PGs to distort their next episode so that theft becomes borrowing. Therefore, more gambling becomes an attempt at reparation; chasing becomes not only revenge against the machines but the hope to repair their personal relationships and their self-esteem. It is much more than what a win can actually achieve.

Sharon observed her personality changing as she began to steal money and eventually lost her job in pursuit of the mythical win that would make everything better. Interestingly, she was aware of her chances of winning, and yet continued to ignore this, engaging in risky behaviours to obtain money to relapse, as she tried for the mythical win that would enable her to win back her losses:

“The sort of person I had become, the gambling, even though I could say myself ‘I can stop. I’ll stop next week.’ Then it escalated because I’d stolen so much money, how will I get that back? Gamble it back. Even though I knew the statistics, the number of wins, I chose to ignore it. That choice is—just that choice is why—whether it was my self-esteem or not, I don’t know. I’m going through a legal issue because I’ve stolen the money. Back when the money stolen was discovered and I was dismissed from work” (Non-Treatment Seeker I, aged 38).

Sharon fantasied about winning $2000 and how good that would feel because she could pay back some of the money she had stolen. This belief enabled her to continue to steal money justifying this behaviour by reassuring herself the mythical win would fix all her problems. This allowed her to ignore the reality of her situation:

“If I did leave with $2000 in my purse, that would feel good. It would be tainted because I won that amount of money from stolen money but then in my head I justified that I will give them half of that money back so it’s like not really with stolen money because I’m giving it back so it was only borrowed money, but I kept borrowing that much and I only gave that much back so it sort of escalated. I would fool myself, justification a hundred times over, of—if I walked out and lost I would think well—there goes all the options again ‘well you can stop, you can wait for your next pay and try and get that back’ or just a lot of things would go through your head. You’d ignore a lot of the bad things because you’d feel too bad so I chose to ignore them” (Non-Treatment Seeker I, aged 38).

### 3.7. Relapse in Fantasy

The process of relapse in fantasy is in fact a “tension-reduction reward” that becomes conditioned into what our participants described as the “merry go round”. Therefore, “relapse in fantasy” occurred when money was lost so the gambler became preoccupied about the next payday and planning their next gambling session while waiting for the money to come into their bank account, so they could gamble finally.

For example, Brenda would plan her next relapse, organising in her mind how she would gamble the night before she got paid. At this time, she admitted she decided to gamble:

“I’d thought about it the night before and thought ‘well, I’ll have time. I’ll go a bit earlier, and I’ll call in’ so I’d virtually organised it in my mind that I would go. I knew I was going to go, and I did so I couldn’t even go straight to my parents’ place, I had to take this bypass to the casino. Yeah, payday was a trigger. The night before would be a trigger because I’d already planned what I was going to do” (Pokies Anonymous member: I, aged 57).

Nick planned his relapse in his mind waiting patiently for his pay to go into his bank. Once his salary was in his bank the “merry-go-round” continued as he lost his money once again:

“You’d be ringing up the bank to see if your money’s in there and as soon as it’s in there, you take off like a rocket. ‘Your beauty, I’ve got money in the bank, I’m going to get all that money back that I lost’” (Pokies Anonymous Member: I, aged 65).

### 3.8. Suicide as an Option to Get Off the ‘Merry-Go-Round’

Some participants reached a point at which they could not endure being caught up on the gambling “merry-go-round”. For these people, death seemed the only option at the time (see Figure 1; Red Circle). For instance, Nanette realised that she had over-stepped the mark with her gambling when she was at her lowest point, and it was at this time that Nanette thought that it would be better for everyone if she were no longer around, as she couldn’t stop relapsing:

“Hitting the lowest. I knew that I’d overstepped the mark to the point where I’d be better off—it’d be better off for everybody if I wasn’t there any longer. I had the tablets in my hand, and I was going to take them” (Pokies Anonymous Member: I, aged 65).

When Simon finally managed to stop gambling, he was able to regain critical thought; however, as he realised the harms associated with his gambling, he immediately contemplated suicide. Once he gained his composure, he realised it was time to stop gambling and reach out for help:

“Well, I was ready to commit suicide, to be honest, and I thought ‘Well, I’ve just got to. If I can’t even do that properly then somehow or other there’s a reason and I have to now stop, and I have to get help to try and stop’” (Treatment Seeker: I, aged 33).

Nick was so distressed at how he had gambled his money that it made him realise how people in a similar situation would go to the extremes of taking their own life when the consequences of gambling were too hard to endure:

“I would come home at night time and say to myself what a fool I was, ‘How stupid can you be losing all that money?’….Very disappointing and that then explains why people rob their bosses, commit suicide, because they can’t live with themselves” (Pokies Anonymous Member: I, aged 65).

### 3.9. Vacillation about Change

The “merry-go-round” is a pattern of learned behaviour that is almost impossible to deal with, resolve or get away from particularly without support. This may be responsible for the huge delay in getting appropriate treatment or quitting. For example, some use the excuse of deferring the decision to gamble with the promise to gamble at a later time: Annie explained that she did not want to recognise that her gambling behaviour was a problem, so she convinced herself that she could stop gambling ‘tomorrow’, but realised that she was powerless to stop the relapse process, and could not see a time when this would occur:

“You sort of don’t want to recognise that you do have a problem and I don’t know where the time for anybody would be where you recognise that you have a problem because you’re so much in denial. Even if you do recognise you can stop tomorrow, but you can’t, and that’s at that point where you can’t stop” (Pokies Anonymous Member: I, aged 53).

Simon also admitted that when he managed to abstain, he thought about the next relapse and planned for a time when he would have the opportunity to gamble. This demonstrates the complexity of the gamblers mind. For example, Brad was attempting to abstain, but still promised himself a relapse and could not relax in anticipation of the gambling. This suggests that Brad had vacillated about gambling and deferred one day at a time:

“Especially if you’re thinking about it and planning your next day or whatever, you could never relax.” Furthermore, the longer he managed to defer the relapse, the more anxious he would become: “Close to the two weeks, 10 days I start getting antsy, 11, 12, towards the end, its like ‘I’ve got to go’” (Treatment Seeker: I, aged 33).

Some gamblers were upset by a big financial loss and were then able to remain abstinent for a few weeks but then relapsed again. For example, Sharon reflected on the amount of money she gambled and realised that it was ridiculous, so she cut up her ATM card in an attempt to stop her repeated relapsing behaviour. However, when she had a new ATM card, her relapse cycle would recommence again:

“I’d go in there and I’d think ‘oh no, I’ll spend a few dollars’ and spent a few more dollars and I thought “no, this is ridiculous,” so I cut my card up. Then I got another card a few weeks later and then I was still going back there” (Non-Treatment Seeker I, aged 38).

### 3.10. Change Behaviour

Accepting the harms associated with gambling and stopping the “merry-go-round” was important in beginning the change process for many participants. Annie described how she learned to accept the harms of her gambling as she realised it was no longer a harmless social outing:

“It may have been over a period of time where you switched from in your mindset thinking that because you’ve gone quite regularly, just socially, and maybe over a couple of weeks, say, then you sort of think ‘Jeepers, I’ve gone there four times out of the couple of weeks and every time I’ve lost’” (Pokies Anonymous Member: I, aged 53).

Participants began to recognise the accumulation of harm that fuelled ongoing relapse and became determined to address their gambling behaviour. Some described a process of acknowledging that their gambling was causing significant problems and reached out for help. Support from significant others helped the gambler begin to address problematic gambling behaviours. With encouragement from another peer, Veronica was able to commit to starting the change process:

“I just rang up, and she told me about the meetings and that. I said ‘Okay, all right. See you,’ and she goes ‘Hang on, are you coming?’ and I go ‘Yeah,’ and because I said ‘Yeah’ I thought – like I’m the type of person that if I say something, I’ll do it, so I made that commitment to say that I would come” (Pokies Anonymous member: I, aged 42).

### 3.11. Determined Not to Gamble Again

Participants described a pattern of gaining insight into their gambling behaviour and associated harms. It was at this time that the participants became determined not to gamble again. However, this was difficult for many, who eventually returned to the gambling “merry-go-round”. Simon described a cycle of gambling where he could abstain from gambling for a few weeks at a time. He admitted that eventually he would return to gamble again chasing a win, and the “merry-go-round” continued:

“Feeling like you’re in a cycle where the more you put in, something’s going to come out. Because there’d be weeks where I’d be okay and then I’d think I was okay and could go and put a bit in and then that’s when the cycle would start again” (Treatment Seeker: I, aged 33).

Leigh realised that, to remain abstinent, he could no longer go into a gaming venue:

“The only way you can control it is not to participate in it, and I guess that describes me with pokies because I cannot participate in it because I cannot walk in” (Pokies Anonymous member: FG, aged 60–65).

### 3.12. The Importance of Support

Eventually, participants realised they needed assistance to leave the “merry-go-round” as they could not stop gambling on their own. For example, Nanette reached out to her son for help when she realised, she had had enough and wanted to stop gambling. He understood that he could not offer her the support she needed so advised her it was time to seek professional help:

“He came in because he had a key to the door and he said ‘What’s the matter?’ and I said ‘I just can’t carry on like this, I need help’ and he said ‘Well, mum, I can’t do it for you, you have to ring and get help’” (Pokies Anonymous Member: I, aged 65).

Annie found reaching out to a 12-step support program for peer support and seeking help from a higher power helped her to break free of the gambling “merry-go-round”:

“I couldn’t listen to myself, but with a higher power, it just gave me an extra, solid strength. So, the 12 steps helped with that, and I was just blown away by these amazing people that are actually reaching out for help. That gave me such a boost I thought—because initially, I was full of judgment when I walked in. I was like ‘Oh my God, what am I doing here?’ and then by the end of that evening I just thought ‘These people are it, they’re doing something’” (Pokies Anonymous Member: I, aged 53).

Nick a Pokies Anonymous member admitted that, with the support from peers, he was able to think rationally about the harms he was causing by gambling:

“I couldn’t rationalise it when I was playing. I can now. This is what we often say at the meetings, where the meetings have helped me it’s enabled me to put these things into perspective because when we were playing, I didn’t put anything in perspective” (Pokies Anonymous Member: I, aged 65).

### 3.13. Recovery

For some, evidence-based treatment provided an exit from the “Merry-go Round” (see Blue Circle Figure 1) When the urge to gamble was fully extinguished, the participants described gaining mastery over their gambling problems and were able to participate in normal living without the ongoing risks of relapse. This was confirmed by their significant others. One treatment seeker reported the exposure treatment he completed had enabled him to “kill” the urge to gamble where past gambling triggers no longer caused him to have an urge to gamble:

“It has totally killed everything; I have no connection” (Treatment seeker: FG: aged 55–65).

This participant’s significant other confirmed that her husband had mastered his problem gambling behaviour and was no longer at risk of relapse:

“The way this course [urge exposure] is structured, he doesn’t have a problem with them anymore” (Significant other: FG: aged 55–65).

Another participant echoed this positive achievement as she had also overcome the urge to gamble and was proud of her achievements:

“There is not even a coping mechanism for me; I no longer have that urge” (Treatment seeker: FG: aged 55–65).

### 3.14. The ‘Merry-Go-Round’ of Relapse

There is a complex interplay of mutually reinforcing, and conflicting cognitions and emotions maintained when the PG receives actual money. For some, the cycle is maintained by them focusing on money in a fantasy until they can source actual money. The process of repeated relapse appears to provide the gambler with an escape from experiencing the despair associated with the harms associated with their gambling and at this time self-observation is not possible, therefore the PG is unable to learn from the harms as a result of their gambling. However, for some, the realisation of harms can slowly begin as they resolve to abstain: Figure 1 shows this as a non-linear process of learning over time. Clearly some PGs die [6] some panic and abstain, recovering spontaneously, some seek help and get better, some remain stuck on the merry-go-round. It is our view that this conflictual process of learning prolongs recovery and leads to drop-out during treatment.

### 3.15. Before Habitual Relapse

After each relapse episode, the PG experienced a temporary period of critical thinking that, for some, was only momentary (Blue Circle Realisation). At this time, a return of critical thinking resulted in the PG being faced with the realisation of the consequences of their behaviour. This realisation and insight created a significant negative affective state, which was extremely distressing for the PG as they acutely experienced significant guilt, grief, and shame associated with their relapse. For the PG to move from the “merry-go-round” into recovery, hope had to outweigh the belief in the “mythical win”, or this belief had to be suspended or deferred, and this seemed much more possible when practical support was offered as seen above. Also, some PGs experienced severe anxiety (Green Circle: Panic) about how they would deal with their desperate financial state which, for most, was intolerable.

As the PG realised the extent of what they had done yet again, their distress often escalated, resulting in suicidal feelings as a means to escape from this desperate situation (Red Circle Figure 1). None of the subjects in this study saw this as an immediate solution; however, many described suicidal despair. The literature indicates that problem gambling is associated with increased suicide risk [20] and clearly, some PGs choose this option, and several participants in the current study described it very clearly, for example: “I’d pinch my husband’s credit card and, of course, it wasn’t coming out my savings, it was coming out of the bank, so I was putting us further and further in debt, and it made me feel absolutely sick. In fact, I did try to commit suicide twice” (Greta: Treatment Seeker: I, aged 69).

The choices at such times appeared to be very stark indeed: one where the PG tried to remain abstinent out of shock or fear; a second, where the PG gives in to the despair in her/his life and decides to suicide; the third and most common choice is that the PG tries to heal this despair by starting their next relapse almost immediately after the last has been completed by fantasying about the next episode when the win could come true. A final option that appears to take many gamblers such a long time to learn is to stop the process of relapse by facing their despair, responsibility, and loss by seeking help and support to assist them to exit this desperate way of living.

The importance of support has been highlighted and should not be underestimated. For some PGs, there is a gradual process of learning from the harms of relapse, which is enhanced if positive support is in place. Support enables the PGs to obtain relief from their negative emotional states, to accept responsibility, and to experience the pain by being genuinely contrite with the significant other and resolving to abstain and not relapse to gambling. In order to do this, the capacity to maintain critical thinking, involved in learning, are required.

If the PG can learn to tolerate their distress, they could then begin to acknowledge the impact of habitual relapse (Blue Circle: Realisation). At this time, they could engage in self-observation where a process of learning from harms (centre of diagram) of their behaviour begins. At this time, the PG tries to remain abstinent and may start to engage in the change process towards recovery actively. Learning to experience emotional states and engage in self-observation did not happen all at once but was built upon and shaped by the PG’s motivation to continue to be vigilant, rather than escaping back to relapse in fantasy until the opportunity to relapse occurred in reality.

### 3.16. Figure 1 ‘Merry-Go-Round’

This diagram shows the complex interplay of mutually reinforcing and conflicting cognitions and emotions maintained when the PG receives actual money.

## 4. Discussion

This study explores the process of habitual relapse in PGs, which provides an understanding as to why PGs continue to relapse despite the harms they must endure. The data were derived from a representative group of PGs, their significant others, and those involved in treatment and gambling help services and have high face validity.

We have postulated a cognitive-behavioural sequence that comprises habitual relapse. When the consequences of their gambling confront the PG and immediately feels despair, the next relapse cycle begins, which reinforces the ongoing cycle of relapse. The PG believes that gambling will offer a “mythical win”, with the fantasy that “all will be right again”. These erroneous cognitions start the next relapse, in fantasy which can be enacted once the money becomes accessible and the venue available.

This explanatory paradigm has high face validity in that parts of these behaviours have been described in many clinical studies, but they have not previously been articulated as a process of habitual relapse. The tension-relief conditioning resulting from the decision to once again believe in winning is what makes this relapse process habitual, and because it habitually relieves the pain of loss, it interferes with the capacity to learn from the loss experience. The PGs’ avoidance of the despair associated with their gambling harms may contribute to the PGs dropping out from professional help due to being traumatised by the comprehensive assessment processes used by clinicians when a PG seeks help. The avoidance of experiencing gambling harms may increase the delay in presentation for treatment or help-seeking. The strength of the qualitative methodologies used has allowed us to propose this model for examination.

It expands the “push” and “pull” models that were proposed following the focus group studies alone [33,34] adding a level of depth and complexity. Problem gambling is a phenomenon that occurs over time and these cumulative experiences of pain, excitement, despair and relief appear to create contradictory behaviours and fluctuating “acts of the will to control playing” that seem to be undone repeatedly by excitement-driven beliefs that are enacted when the PG is in a different frame of mind or cognitive set, when memory function is suspended and “contrary acts of the will are permitted and actively facilitated”. These emotions and cognitions involve the limbic system, the prefrontal cortex and memory circuits. This suggests that complex neuro-circuits are shaped by the complicated tension-relief neural rewards [35], which perhaps fit the criteria required in the process of dissociation, as memory is clearly impaired during this habitual “merry-go-round” As such, this methodology has pieced together a purported process that needs to be verified by replication, using alternative methodologies including quantitative measures. These findings should be examined in other communities, for other forms of gambling, regular gamblers, in those who occasionally use EGMs or during the development of problem gambling in order to test whether these findings withstand such scrutiny. Also, the use of purposive sampling means the findings may not be generalizable given that data was gathered from individuals in a non-random manner and from volunteers who received payment for expenses.

### 4.1. Implications and Further Research

If ‘habitual relapse’ is triggered when PGs experience an intense negative affective state, the therapeutic process of exploring the impacts of harmful gambling in the PG’s life may result in them “feeling bad” while making them “take responsibility” for their behaviours, and this may be too distressing for some. This may be the reason for PGs dropping out of treatment early or not attending appointments. Therefore, the entire assessment process could be producing an episode of habitual relapse, a point when many patients drop out of the therapeutic program.

Treatment drop-out could also happen at any point of the therapeutic process when a counsellor, financial adviser; a 12-step meeting or a treating CBT therapist is working with a PG, and they are dealing with painful events or issues resulting from previous gambling episodes in therapy; again “relapse in fantasy” can result so the patient will drop-out of treatment. Furthermore, the ease with which relapse in fantasy can be triggered needs to be considered during the assessment and addressed as part of the harm minimisation strategy.

Therefore, consideration needs to be taken to ensure that assessment and treatment are progressed in a manner that allows the PG to acknowledge the harms associated with their behaviour gradually. Therefore, a mental health assessment needs to be carried out with all PGs [36] as many PGs suffer from depression or negative affective states, as described by many participants in this study. The mental health assessment could include the recognition and management of all co-morbidity, especially depression, which has been highlighted in this study as a significant risk factor for habitual relapse. Perhaps when patients do miss appointments, follow up is required to check on relapse in fantasy. Reducing drop-outs is a critical issue [37].

Finally, some PGs may already be in “relapse in fantasy” mode when they attend professional help as they are already planning the next relapse. At this time the PG may well not be aware that this is happening. At this time, learning to address the issues necessary for recovery may become too complicated. These PGs may not be ready to address their gambling problems and may find that planning their next relapse helps to distract them from experiencing the distress needed to address their gambling problems and to begin to recover. Part of any assessment needs to take this into account.

At this time, support is essential to provide a safety net that enables the PG to experience this distress while also keeping them from becoming so distressed that they feel like committing suicide or relapsing into gambling. However, further research is needed to explore and understand what “effective support” actually consists of that will enable these PGs to experience the distress of acknowledging the harms of their behaviours. This support will help the PG to progress through the learning process to begin to recover.

### 4.2. ‘Merry-Go-Round’ Model

The model needs to be examined further to understand the following findings:

Suicidal despair leads to suicide as one possible outcome.

In order to protect the self from suicide, “relapse in fantasy” is a lesser evil that is protective at this time, so most PGs choose this immediate solution.

The anxiety and guilt associated with problem gambling behaviour also lead many to resolve to abstain, and they do not relapse in fantasy but are vulnerable to relapse at any time.

Realisation allows the PG to seek help and acknowledge the severity of the personal problem rather than just the external problems, seek support, and make the decision to seek a good solution.

For PGs, this merry-go-around can perpetuate a range of intergenerational harms. Even for those PGs who are able to leave this merry-go-round, the accrued financial harms can be significant from a legacy perspective impacting on the PG’s life course leading to intergenerational harms, on both the gambler and their affected others. For example, the PG may experience poor credit ratings, financial vulnerability, poverty and homelessness. Other intergenerational harms include relationship conflict or breakdown, job loss, incarceration, or child neglect where children are removed from the person’s care [38]. Furthermore, when a significant other witnesses someone close to them such as a parent engaging in gambling or discussing possible gambling wins this can create outcome expectancies that serve as positive incentives for this person to gamble [39]. This role modeling can impact on the development of gambling problems and ongoing harms between generations. Therefore, the results of this study make a convincing argument that PG, and the merry-go-round that is a strong feature of gambling relapse, is a significant public health issue. This highlights the importance of the role that health professionals can play in identifying and treating this problem, which can cause substantial harm to the individual and the community.

## 5. Conclusions

In the development of this model three conceptual changes in respect of relapse have been postulated more than is extant in the current literature, and there is a greater complexity of the interplay of factors involved in repeated relapse in comparison to current theories about awareness and avoidance of risk situations, cue exposure, and other basic actions that the person could/should take [17]. For example: a “mythical win” (the win that will make everything better again) as distinct from any win; “relapse in fantasy” with the deferral of enacted relapse until “available money” becomes “accessible” [40] and “habitual relapse” develops. These three conceptual developments clearly need consideration, review and testing to see whether they have utility in understanding relapse and treatment of EGM problem gambling. A limitation of this study is that the gambling participants recruited for this study had self-identified as gamblers, rather than being identified by fulfilling criteria through diagnostic screening tools.
